# Exceptional Financial Support for Introduction of Inactivated Polio Vaccine in Middle-Income Countries

**DOI:** 10.1093/infdis/jiw573

**Published:** 2017-06-30

**Authors:** Anne-Line Blankenhorn, Tania Cernuschi, Michel J. Zaffran

**Affiliations:** 1 Independent consultant, Geneva, Switzerland;; 2 Extended Programme on Immunization, and; 3 Polio Eradication, World Health Organization, Geneva, Switzerland

**Keywords:** financial support, middle income countries, vaccine introduction, inactivated polio vaccine, polio endgame.

## Abstract

In May 2012, the World Health Assembly declared the completion of poliovirus eradication a programmatic emergency for global public health and called for a comprehensive polio endgame strategy. The Polio Eradication and Endgame Strategic Plan 2013-2018 was developed in response to this call and demands that all countries using Oral Polio Vaccine (OPV) only introduce at least 1 dose of Inactivated Polio Vaccine (IPV) into routine immunization schedules by the end of 2015. In November 2013, the Board of Gavi (the Vaccine Alliance) approved the provision of support for IPV introduction in the 72 Gavi-eligible countries. Following analytical work and stakeholder consultations, the IPV Immunization Systems Management Group (IMG) presented a proposal to provide exceptional financial support for IPV introduction to additional OPV-only using countries not eligible for Gavi support and that would otherwise not be able to mobilize the necessary financial resources within the Polio Eradication and Endgame Strategic Plan timelines. In June 2014, the Polio Oversight Board (POB) agreed to make available a maximum envelope of US $45 million toward supporting countries not eligible for Gavi funding. This article describes the design of the funding mechanism that was developed, its implementation and the lessons learned through this process.

## OVERVIEW

Objective 2 of the Polio Eradication Endgame Strategic Plan 2013–2018 calls for the introduction of at least 1 dose of inactivated polio vaccine (IPV) into routine immunization schedules by the end of 2015 before the withdrawal of type 2 oral polio vaccine (OPV) as the first step toward the complete withdrawal of all OPV by 2020 [1]. IPV introduction was to take place by the end of December 2015, 6 months prior to the withdrawal of OPV type 2 serotype (OPV2) planned in April 2016. IPV would ensure a substantial proportion of the population was protected against the type 2 polio serotype once OPV2 was withdrawn, to boost population immunity to types 1 and 3 serotypes [2] and to accelerate efforts toward polio eradication.

The introduction of IPV required comprehensive coordination to ensure that an unprecedented number of countries could introduce a new vaccine in their routine immunization program within a tight timeline. Any delay in introduction could jeopardize the effective withdrawal of OPV2 planned in 2016. Global and regional immunization stakeholders organized themselves through an Immunization Systems Management Group (IMG) of the Global Polio Eradication Initiative (GPEI), responsible for the management and coordination of partner activities to achieve Objective 2 of the Polio Endgame Strategic Plan. The IMG worked to ensure countries had access to the resources needed to plan and effectively introduce IPV, while accounting for competing priorities such as other planned new vaccine introductions.

A total of 126 countries only using OPV were concerned with IPV introduction at the time of planning in June 2013. In November 2013, the Board of Gavi (the Vaccine Alliance) approved the provision of financial and technical support for the introduction of IPV in the 72 OPV-only using Gavi-eligible and -graduating countries. By June 2014, 84% of these countries had developed a plan to introduce IPV within the Endgame timelines [2], and by January 2015, 71 of the 72 Gavi-eligible countries had submitted an application for support to IPV introduction [3]. The first Gavi-eligible country to introduce IPV was Nepal, in September 2014 [4].

In stark contrast to such achievements, the remaining 54 OPV-only using countries not eligible for Gavi support were progressing much more slowly, with only 11 of 54 countries having introduced (n=4) or formally committed to introducing (n=7) IPV as of June 2014. These countries formed a highly heterogeneous group in terms of size, geography, political stability, and socioeconomic characteristics, with country annual birth cohorts ranging from 153 people to 18 million and gross national income (GNI) per capita ranging from US$2470 to US$14400, according to analyses conducted by the World Health Organization (WHO) at the time. Aside from the 4 non-Gavi countries that had already introduced IPV, the remaining 50 countries included 10 lower middle-income countries (LMICs) and 40 upper middle-income countries (UMICs), according to the World Bank classification at the time. Given slow progress, GPEI partners began to express concern with potential delays in introduction. Similar concerns vis-à-vis the pace of new vaccine introductions in middle-income countries (MICs) had already been experienced with other vaccines such as *Haemophilus influenzae* type b, hepatitis B, and pneumococcal and rotavirus vaccines [5]. Causes for slower progress with vaccine introduction included a lack of funding, weak national decision-making processes, and regulatory pathways. The international community seemed to agree that in the case of IPV, difficulties in mobilizing sufficient national resources could jeopardize the accelerated timelines set through the Polio Endgame Strategic Plan. In addition, the affordability of IPV vaccines seemed to be an issue, as well as system readiness considerations.

## THE MIDDLE-INCOME COUNTRY ISSUE

The idea of extending support to countries above Gavi’s GNI per capita eligibility threshold (corresponding at the time to a GNI per capita of US$1500 updated annually to account for inflation [6]) for introduction of IPV were met with strong concerns among the donor community. Indeed, public health and, more generally, global development assistance has increasingly prioritized countries deemed to be lagging furthest behind and with less resources to provide/care for their populations [7]. In contrast, MICs are generally considered to have stronger institutional capacities as well as access to further resources, and thus to be in lesser need for external assistance [8]. A review of aid directed toward MICs shows a progressive decline of assistance to MICs from the early 1990s [9]. The share of overseas development assistance (ODA) received by the 2011 MIC cohort fell from circa 55% in 1990 to under 40% per most recent World Bank data from that year [10]. Furthermore, in 2012, the average net ODA received as a percentage of GNI was close to 10% among lower-income countries (LICs), 1% among LMICs, and close to 0% among UMICs, and the net ODA received as a percentage of gross capital formation averaged 40% among LICs, 3% among LMICs, and close to 0% among UMICs. Looking at traditional donors, the share of aid provided to MICs by Canada and the Netherlands was reduced by one-third between 1998–1999 and 2008–2009, and by one-quarter by the United Kingdom, Norway, and the United States over the same period [9]. However, “nothing automatically happens when a country crosses a line in per capita income. … [although] it appears that as countries grow and aid becomes less significant as a proportion of GNI, OECD donors find it increasingly hard to defend aid to them; they are no longer ‘the poorest countries in the world’” [10].

The IMG presented the donor community with different arguments supporting the extension of assistance to MICs for IPV introduction. First, the “poverty-reduction argument”: While many LICs transitioned to MIC status following rapid economic growth over the last decade, this growth was not accompanied by expected levels of poverty reduction [11]. Consequently, the distribution of the world’s poor simply shifted toward MICs, and 70% of the world’s poor now live in these countries [8]. This is an important change from 1990, when 93% of the world’s poor lived in LICs and could thus be reached by development assistance targeted to the poorest countries [12, 13]. Second, the “disease burden argument”: Although MICs with the highest shares of vaccine-preventable diseases and unvaccinated children are currently eligible for support by Gavi, MICs that are not eligible represent an important share of unvaccinated children and preventable morbidity and mortality [8, 14]. Certainly, no disease can be eliminated or eradicated if some countries are left behind. Thus, the “all-or-nothing” approach to ODA for immunization would likely lead to the failure of polio eradication efforts through the Endgame Strategic Plan. Last, while it was recognized that MICs boast relatively strong immunization systems, the country consultation process conducted by the IMG documented weaknesses in planning, budgeting, financing, and procurement processes, and thus justified the need for exceptional support to respond to a global health emergency such as the introduction of IPV.

Donors acknowledged the risk of delayed IPV introduction, yet voiced strong concerns around setting a precedent around MIC funding. Any support to these countries would need to be time-limited, catalytic, and focused on countries most at risk of delayed IPV introduction and most in need of support. Donors thus accepted limited and exceptional support, but asked that eligibility be structured in a manner aligned with country risk and financial need, with support provided on a case-by-case basis as a last resort. Also, support would need to be provided on the explicit condition that the country would commit to take over the financing of the vaccine after the first year of GPEI support.

These recommendations formed the basis for the grant-making model developed by the IMG to provide financial support to select non-Gavi OPV-only using countries for IPV introduction. The IMG’s proposal focused on selecting countries by combining the risk of polio outbreak (as determined by country “tiers”) with financial need as determined by GNI per capita. [The IMG established criteria to identify countries at the highest risk of a type 2 circulating vaccine-derived poliovirus outbreak and importations following OPV2 cessation, categorizing countries within “tiers,” with Tier 1 countries corresponding to those most at risk of a polio outbreak and Tier 4 countries to those at least risk. Thus, countries deemed eligible to receive funding were LMICs as well as Tiers 2 and 3 countries that had not yet introduced IPV.] Applying these criteria, a total of 16 countries (Cape Verde [Tier 4], Dominican Republic [Tier 2], Arab Republic of Egypt [Tier 3], Republic of El Salvador [Tier 4], Equatorial Guinea [Tier 2], Gabon [Tier 2], Republic of Guatemala [Tier 4], Republic of Iraq [Tier 2], Kingdom of Morocco [Tier 4], Republic of Paraguay [Tier 4], Republic of the Philippines [Tier 2], Independent State of Samoa [Tier 4], Swaziland [Tier 4], Turkmenistan [Tier 3], Republic of Vanuatu [Tier 4], and Iran [Tier 4]) were deemed eligible to receive support through this mechanism across the WHO African, American, Eastern Mediterranean, and Western Pacific regions, representing a birth cohort of 8.7 million (per United Nations Development Programme [UNDP] 2012 data) [15]. Among these countries were 10 LMICs, 5 UMICs, and 1 high-risk Tier 2 high-income country (HIC) (Table 1).

**Table 1.** Application of Polio Risk and Financial Need Criteria to Select Countries Eligible for IPV Introduction Support

Country	Income Group	WHO Region	Birth Cohort (UNDP 2012)	WB 2012GNI Per Capita	IPVRisk Tier
Cape Verde	LMIC	AFRO	10132	3810	4
Dominican Republic	UMIC	AMRO	217671	5470	2
Egypt	LMIC	EMRO	1898349	3000	3
El Salvador	LMIC	AMRO	127614	3580	4
Equatorial Guinea	HIC	AFRO	26422	13560	2
Gabon	UMIC	AFRO	52664	10070	2
Guatemala	LMIC	AMRO	474434	3120	4
Iraq	UMIC	EMRO	1036907	5870	2
Morocco	LMIC	EMRO	738722	2940	4
Paraguay	LMIC	AMRO	160147	3290	4
Philippines	LMIC	WPRO	2382822	2470	2
Samoa	LMIC	WPRO	5058	3220	4
Swaziland	LMIC	AFRO	37134	2860	4
Turkmenistan	UMIC	EURO	111345	5550	3
Vanuatu	LMIC	WPRO	6659	3080	4
Iran	UMIC	EMRO	1454039	(2009) 4330	4

Abbreviations: AFRO, WHO Regional Office for Africa; AMRO, WHO Regional Office for the Americas; EMRO, WHO Regional Office for the Eastern Mediterranean; GNI, gross national income; HIC, high-income country; IPV, inactivated polio vaccine; LMIC, lower middle-income country; UMIC, upper middle-income country; UNDP, United Nations Development Programme; WB, World Bank; WHO, World Health Organization; WPRO, WHO Western Pacific Regional Office.

## DESIGN OF THE FUNDING MECHANISM

In-depth analytical work (including an ability to pay model, a willingness to pay survey, and consultations with WHO regions) as well as consultations with partners, countries, and donors (including discussions through IMG financing and implementation subgroups, discussion with major IPV donors on 9 May 9 2014, and discussion with the Polio Partners Group on 16 June 2014) were conducted to design the funding mechanisms for IPV introduction in the selected countries. This was a Gavi-like, yet lighter touch, mechanism, combining a vaccine introduction grant (VIG) with time-limited support for IPV supply through the United Nations Children’s Fund (UNICEF) Supply Division.

The VIG component was designed as a one-time grant aiming to offset the operational costs of the new vaccine introduction, that is, to support costs that are directly related to routine program implementation such as costs relating to social mobilization and advocacy efforts for the program, its human resources, the procurement and maintenance of cold chain equipment, or those associated with transport and supervision. The determination of the financial envelope needed by countries for these purposes was informed by a secondary review of system costs of introducing a new vaccine in MICs, conducted by the Task Force for Global Health on behalf of the IMG in February 2014 (unpublished). As a result of these findings and in line with Gavi’s new vaccine introduction financing thresholds, the IMG concluded that a VIG amounting to US$0.80 per child in the birth cohort or a minimum of US$100000 would adequately cover the operational costs associated with IPV introduction.

The initial procurement support component aimed to provide countries with 12 months of IPV supply through the UNICEF Supply Division to ensure they could meet Endgame timelines, regardless of budget cycles and of the availability of funds to support this activity. As is the case in the Gavi model, the procurement cost estimates were based on modeled estimates of country birth cohorts provided by UNDP and diphtheria-tetanus-pertussis immunization coverage estimates and projections. The costing for this component also varied according to vaccine presentation, accounting for vial size and associated wastage rates. The price of the vaccine accessible through this mechanism was guaranteed through a UNICEF-specific tender, allowing countries to access the same price as Gavi countries for the 5-dose vial and the range of prices set through Sanofi for the 10-dose vial according to income category.

Initial budgetary estimates that combined both the VIG and the procurement components suggested financial requirements of up to US$45 million were necessary to enable IPV introduction in most at-risk MICs. The POB approved the IMG’s proposal in June 2014, agreeing to make this amount available as a maximum ceiling toward the goal of accelerated IPV introduction in the selected countries. (US$1 million of these funds was set aside for the management of the funds disbursement process.)

To ensure that the provision of financial support to each of the eligible countries would be made on a case-by-case basis and based on demonstrated country need, the IMG proposed to leverage the capacities of the WHO regional offices and asked countries to submit an application to WHO regional colleagues for screening. Meanwhile, a working group responsible for launching and managing the application and grant-making process was established, formed of representatives from WHO and UNICEF HQ and regions, as well as the Bill & Melinda Gates Foundation. This working group developed detailed and comprehensive guidelines and standard operating procedures to guide the country application process, including objective criteria to screen and validate these applications. Such criteria mainly focused on: (1) the completeness of country applications and vaccine introduction plans, (2) the adequacy of the proposed IPV introduction date with Endgame timelines, (3) the availability of supply in the requested product presentation at the UNICEF SD level, and (4) the country’s commitment to financial sustainability, as demonstrated by the submission of a formal letter that indicated its pledge to the continued financing and supply of IPV beyond GPEI support, signed by the Ministry of Health or Finance.

It was agreed that upon review of complete applications by the Ministries of Health of eligible countries and a dialogue involving both WHO and UNICEF country and regional offices, the working group would discuss country requests via teleconference and/or email communications, and made recommendations to the IMG for final approval within 1 working week. The working group would then trigger the disbursal of VIG funds via WHO headquarters to WHO country offices for onward disbursement to Ministries of Health and/or direct implementation to where the funds were needed and requested by countries, and confirmed the procurement of IPV doses to UNICEF SD.

## THE FUNDING MECHANISM IN ACTION

Upon opening the funding stream in December 2014, it became apparent that a number of additional countries were at risk of missing the global target of introducing IPV by the end of 2015, due to insufficient levels of funding mobilized for IPV introduction. Three countries in WHO’s Pan-American region (Belize, Ecuador, and Jamaica) and 6 in the Western Pacific region (Cook Islands, Nauru, Fiji, Tokelau, Tonga, and Tuvalu) submitted official requests for support, and the POB subsequently approved the extension of funding to these 9 additional countries for an amount of US$ 1183299. Although these countries were UMICs and categorized as Tier 4 by GPEI due to their relatively low risk for a polio outbreak, they all faced specific, complex economic and political challenges that made a rapid new vaccine introduction difficult.

Following the POB’s approval, 25 MICs were eligible to receive support through the mechanism (Belize, Cabo Verde, Cook Islands, Dominican Republic, Ecuador, Arab Republic of Egypt, El Salvador, Equatorial Guinea, Fiji, Gabon, Guatemala, Iran, Iraq, Jamaica, Morocco, Nauru, Paraguay, Philippines, Samoa, Swaziland, Tokelau, Tonga, Turkmenistan, Tuvalu, and Vanuatu). Of these 25 countries, 7 (Cook islands, Morocco, Nauru, Tokelau, Tonga, Tuvalu, and Iraq) did not proceed with submitting an application due to varying reasons, including: (1) the availability of sufficient domestic resources to support IPV introduction (Morocco), (2) the availability of other donor funds to support IPV introduction (Western Pacific region countries), and (3) a national decision to introduce another vaccine presentation than the standalone IPV (hexavalent vaccine in Iraq). The remaining 18 countries submitted an application and were successful in accessing funding to support IPV introduction. Of note, 10 countries received funds for a VIG only, and 8 countries for both initial procurement support and a VIG.

As a consequence, of the US$ 45 million envelope approved by the POB to support IPV introduction in OPV-only using non-Gavi countries, only US$16 million was needed and disbursed by the close of the funding window in June 2015. The total cost of the VIG was US$7 million, and US$ 9 million for the procurement of close to 5 million doses of IPV as well as freight costs and injection supplies.

An analysis of the budgets submitted by countries as part of their applications suggests that the greatest funding needs faced by countries were in the area of cold chain equipment, with close to 30% of funds disbursed for the VIG towards these activities. A further 20% of the funds were requested for surveillance and monitoring activities and 12% to document production.

Countries who received support successfully introduced IPV into routine immunization schedules by the end of 2015, in line with the global target set through the Endgame Plan [16]. However, due to global IPV supply constraints, vaccine deliveries to countries deemed at lower risk of a polio outbreak were postponed until the final quarter of 2017 [17]. This affected the introduction in 2 eligible countries (Turkmenistan and Egypt). Although these countries were planning and ready to introduce IPV by December 2015, they are now formally committed to introducing IPV by December 2017. A number of other countries, including LICs and MICs supported by Gavi, are in a similar position.

## LESSONS LEARNED

Although no attempt was made to measure the incremental speed of vaccine introduction and its causes, we certainly can say that based on the needs expressed by countries through their applications and confirmed by WHO and UNICEF counterparts, the exceptional funds made available to MICs through this mechanism facilitated the implementation of crucial activities for IPV introduction within a short time frame. Furthermore, it is likely that investments made through this effort (for instance, in strengthening the immunization cold chain) will facilitate the introduction of future vaccines in many of these countries going forward. A number of other lessons can also be learned.

When a global target is set and prioritized and the international community works with each country to meet this target, MICs seem to be able to move at the same speed as countries heavily supported by international aid. Dedicating minimal resources to working with MICs is key to their success. This may involve communicating global goals; landscaping supply, demand, and regulatory matters for all nations; reviewing country-specific obstacles; and providing time-limited and targeted technical and financial support.

This experience also highlighted that time-limited and catalytic support may not foster donor dependence. Indeed, some of the countries eligible for IPV introduction support committed to self-financing, and did not modify these plans once alternative funds became available. Furthermore, countries that had identified funds from other donors to support the introduction did not request further funds to cover other related costs through this mechanism. As a result, the final cost of the scheme represented only 35% of the US$45 million set aside for this scheme, a fraction of the anticipated cost.

When dealing with a very heterogeneous group such as MICs and when managing very limited support, established relationships with countries can be key to success. WHO and UNICEF regional offices can play a crucial role in engaging MICs and ensuring the provision of targeted and meaningful support. WHO and UNICEF regional focal points for immunization and new and underutilized vaccines implementation entered in a fruitful dialogue with eligible countries to identify real needs for external assistance to meet Endgame timelines for IPV introduction, and advised on the specific bottlenecks faced by these countries in accessing or mobilizing the necessary funding. Focal points were instrumental in communicating the exceptional and time-limited nature of the funding mechanism that occurs in the very specific context of the implementation of the Polio Endgame Plan, thus ensuring no further support for immunization was taken for granted by recipient countries.

## CONCLUSIONS

This article provided an overview of the exceptional support offered to a limited number of MICs for IPV introduction in the framework of Objective 2 of the Polio Endgame Strategic Plan. It described how this funding mechanism was designed and the lessons that have been learned through this process. Although these countries have stronger health systems and access to further domestic resources to support their national programs, they also face significant challenges and bottlenecks that may slow down the introduction of new vaccines. While technical and financial support in the area of public health and immunization is almost exclusively focused on LICs through an “all-or-nothing” approach, this article has described how catalytic and limited support in HICs can go a long way toward ensuring progress against global goals and targets.

## Notes

***Supplement sponsorship.*** This work is part of a supplement coordinated by the Task Force for Global Health with funding provided by The Bill and Melinda Gates Foundation and the Centers for Disease Control and Prevention.

***Potential conflicts of interest.*** All authors: No reported conflicts.
